# miR‐4448/Girdin/Akt/AMPK axis inhibits EZH2‐mediated EMT and tumorigenesis in small‐cell lung cancer

**DOI:** 10.1002/cam4.70093

**Published:** 2024-10-14

**Authors:** Nobuyuki Koyama, Yuichi Ishikawa, Hiromitsu Ohta, Takuya Aoki, Hiroyuki Kyoyama, Kazutetsu Aoshiba, Kazutsugu Uematsu

**Affiliations:** ^1^ Department of Respiratory Medicine, Saitama Medical Center Saitama Medical University Kawagoe‐shi Saitama Japan; ^2^ Department of Pathology, School of Medicine International University of Health and Welfare Minato‐ku Tokyo Japan; ^3^ Clinical Department of Internal Medicine Saitama Medical Center, Jichi Medical University Saitama‐shi Saitama Japan; ^4^ Department of Clinical Oncology, Hachioji Medical Center Tokyo Medical University Hachioji‐shi Tokyo Japan; ^5^ Department of Pulmonary Medicine, Ibaraki Medical Center Tokyo Medical University Inashiki‐gun Ibaraki Japan

**Keywords:** Akt, AMP‐activated protein kinase, epithelial‐mesenchymal transition, enhancer of zeste homolog 2, Girdin, miR‐4448

## Abstract

**Background:**

Small‐cell lung cancer (SCLC) shows high enhancer of zeste homolog 2 (EZH2) expressions. EZH2‐mediated epigenetics promote epithelial‐mesenchymal transition (EMT), enhancing invasive and metastatic potential in malignancies. MicroRNAs (miRNAs), small noncoding RNAs, modulate EMT, determining tumor phenotypes. However, the association between miRNAs and EZH2 in SCLC remains to be clarified—we aimed to identify a novel tumorigenic mechanism through miRNAs, EZH2, and EMT in SCLC, leading to future therapeutic applications.

**Methods:**

We analyzed EZH2 and E‐cadherin expressions in lung cancer cell lines and tumor tissues from 34 SCLC patients and confirmed EZH2 siRNA‐mediated EMT inhibition. miRNA expression profiles were compared between EZH2 knockdown SCLC cells and negative control SCLC cells using miRNA array. We identified a target miRNA of EZH2 showing expressional differences in EZH2‐knockdown cells and analyzed the impact of the miRNA on EZH2‐mediated EMT and tumorigenesis.

**Results:**

All SCLC cells showed increased EZH2 and decreased E‐cadherin expressions. SCLC tissues had higher EZH2 and lower E‐cadherin expressions than other lung cancer tissues. miRNA array revealed that miR‐4448 expression increased in EZH2‐knockdown SCLC cells. miR‐4448 overexpression reduced tumor cell growth and prevented EMT. miR‐4448 bound to the 3′UTR of the *girdin* gene and suppressed its expression, thereby decreasing Akt phosphorylation at Ser473. Attenuated Akt phosphorylation resulted in AMP‐activated protein kinase (AMPK) phosphorylation at Thr172 and 183, enhancing EZH2 phosphorylation at Thr311.

**Conclusion:**

SCLC characterized high EZH2 expression and promoted EMT, compared with non‐small cell lung cancer. miR‐4448 inhibited Girdin expression, reducing Akt phosphorylation, and enhancing AMPK and EZH2 phosphorylation. Eventually, miR‐4448 prevented EZH2‐mediated EMT and tumorigenesis by modulating the Girdin/Akt/AMPK axis in SCLC. miR‐4448 might be a potential SCLC inhibitor.

## Graphical abstract

Small cell lung cancer (SCLC) showed high enhancer of zeste homolog 2 (EZH2) expression and promoted epithelial‐mesenchymal transition (EMT), and miR‐4448 prevented EZH2‐mediated EMT and tumorigenesis by modulating the Girdin/Akt/AMP‐activated protein kinase axis. miR‐4448 might be a potential SCLC inhibitor.

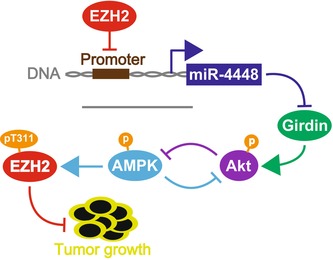

## 1 INTRODUCTION

Lung cancer is the main contributor to cancer death worldwide,^1^ and small‐cell lung cancer (SCLC) covers 10%–20% of all lung cancers. Albeit a multidisciplinary therapeutic approach, SCLC is characterized by poor outcomes due to tumor invasion and metastasis in the early stage and acquired resistance to chemotherapy. To improve SCLC prognosis, we need to elucidate the mechanisms underlying cancer proliferation, invasion, and metastasis.

Previous reports have suggested that the epithelial‐mesenchymal transition (EMT) is concerned with the biological phenotype of SCLC.^2^, ^3^, ^4^ EMT induction is recognized as an early event in cancer invasion and metastasis. In this process, epithelial cells lose polarity and cell–cell adhesion, acquiring migratory and metastatic potentials to be transformed into mesenchymal cells.^5^ Thus, EMT is one of the crucial phenomena for tumor invasion and metastasis as well as immune modification in the tumor microenvironment and autophagy in various tumor types.^6^ EMT modulates signaling pathways, changes in cellular morphology through epigenetic modifications, and gene expressions, including growth factors and cytokines which characterize biological trends and clinical presentation of SCLC.^7^ Clinically, EMT is involved in chemoresistance in patients with SCLC.^8^

Of the EMT‐associated molecules, microRNAs (miRNAs) have been reported to impact EMT regulation.^9^ miRNAs, single‐stranded, noncoding small (18–22 nucleotides long) RNAs, bind to the 3′UTRs of target mRNAs, leading to repressed gene transcription and epigenetic gene regulation.^10^ Epigenetic modification through miRNAs plays a critical role in many biological processes: cellular differentiation, tumor proliferation, cell death, cancer metabolism, and energy homeostasis.^11^, ^12^, ^13^, ^14^ Thus, aberrant miRNA expressions can dysregulate EMT and enhance tumor aggressiveness.^15^

As a crucial component of polycomb repressive component 2 (PRC2) in the polycomb group (PcG) family, enhancer of zeste homolog 2 (EZH2) trimethylates histone 3 lysine 27 (H3K27), resulting in gene silencing through epigenetic modification.^16^ EZH2 regulating embryonic development promotes tumor proliferation, invasion, and metastasis through EMT regulation.^17^, ^18^ EZH2 expression levels are concerned with clinical prognosis in various cancer types.^19^ We previously reported that high EZH2 expression is related to poor outcomes in patients with stage I non‐small cell lung cancer, large tumor size, and enhanced cellular proliferation.^20^ In this context, miRNAs are deemed to fulfill a crucial function in EZH2‐induced EMT and tumorigenesis.^21^, ^22^ The interactions between miRNAs and EZH2 promote tumorigenesis through EMT in different tumors.^23^ Sato et al. reported high EZH2 expression in SCLC.^24^ Gardner et al. showed that EZH2 is involved in the pathogenesis and chemoresistance of SCLC.^25^ However, the association between miRNAs and EZH2 in SCLC remains poorly understood.

To uncover miRNAs associated with EZH2‐mediated EMT and tumorigenesis in SCLC, we performed a comprehensive miRNA expression analysis for EZH2‐knockdown SCLC cells. miR‐4448 was extracted based on the expression data and the previous report.^26^ Furthermore, we investigated how miR‐4448 modulated EZH2‐mediated EMT and cellular proliferation to identify a novel tumorigenic mechanism in SCLC. The current study indicated that miR‐4448 reduced tumor proliferation, invasion, and metastasis through EZH2‐induced EMT, providing a new perspective for designing future therapeutic strategies against SCLC.

## 2 MATERIALS AND METHODS

### 2.1 Patients

A total of 34 patients with SCLC who underwent surgical intervention at Jichi Medical University Saitama Medical Center between 2000 and 2013 were enrolled in the study. Of these, 16 patients were in stage I (47%), 10 in stage II (29%), seven in stage III (21%), and one in stage IV (3%) of SCLC. As a reference, the study included 34 stage‐matched patients, each with adenocarcinoma (AD) and squamous cell carcinoma (SQ). After the institutional review board of Jichi Medical University Saitama Medical Center approved the study (No. 14–13), surgical specimens from 102 patients with SCLC, AD, and SQ were subjected to immunohistochemistry. The institutional review board of Jichi Medical University Saitama Medical Center waived the informed consent for the noninvasive retrospective study. All experiments were implemented following approved guidelines and regulations.

### 2.2 Immunohistochemical analysis

Thin sections of 10% formalin‐fixed paraffin‐embedded tissue specimens were stained with an antihuman EZH2 monoclonal antibody or an anti‐human E‐cadherin monoclonal antibody (Table S1). EZH2 and E‐cadherin expression levels were assessed by the immune‐reactive score (IRS) defined based on the proportion and the staining intensity of cells expressing target proteins.^27^ The proportion of the cancer area stained in high‐power fields was measured. Staining intensity was divided into four grades: 0 (negative), 1 (weak), 2 (moderate), and 3 (strong), and the proportion of positive cells was classified into five grades: 0 (negative), 1 (<10%), 2 (11%–50%), 3 (51%–80%), and 4 (>80%). IRS obtained from the multiplication of these two factors (0–12) was determined as follows: 0, negative; values 1–3, weak; 4 and 6, positive; and multiplication values 8, 9, and 12, strongly positive.

### 2.3 Cell cultures

Lung cancer cell lines from the following sources were employed: NCI‐H1975, NCI‐H1650, PC3, NCI‐H2228 (AD), SBC1, SBC3, and SBC5 (SCLC) were kindly gifted by Dr. Yuichi Ishikawa, the Japanese Foundation for Cancer Research (Tokyo, Japan). RERF‐LC‐AI (SQ) and LC‐2/ad (AD) were purchased from the Riken Bioresource Center (Tsukuba, Japan); Sq‐5 (SQ) and A549 (AD) were derived from the Cell Resource Center for Biomedical Research Tohoku University (Sendai, Japan); and NCI‐H1299 (large cell neuroendocrine carcinoma) and BEAS‐2B (human bronchial epithelium) cells were purchased from the American Type Culture Collection (Manassas, VA, USA). Cells were grown in RPMI‐1640 medium (Sigma‐Aldrich, St. Louis, MO, USA) supplemented with 10% fetal bovine serum (GE Healthcare Life Sciences, South Logan, UT, USA) in a humidified chamber at 37°C under 5% CO_2_.

### 2.4 Quantitative real‐time RT‐PCR (qPCR) for mRNA

Total RNA from each cell line was extracted using the illustra RNAspin Mini Isolation Kit (GE Healthcare, Little Chalfont, Buckinghamshire, UK), reverse transcribed using High‐Capacity RNA‐to‐cDNA Kit (Thermo Fisher Scientific, Waltham, MA, USA), and mixed with SYBR Premix Ex Taq (Takara Inc., Shiga, Japan). The resulting cDNAs were amplified via polymerase chain reaction (1 cycle at 95°C for 120 s, 40 cycles at 95°C for 5 s, and 60°C for 30 s) using the QuantStudio™ 12 K Flex Real‐Time PCR System (Thermo Fisher Scientific). The primers used are listed in Table S2. The amounts of *EZH2* and *E‐cadherin* mRNA were normalized to the amount of *GAPDH* mRNA.

### 2.5 EZH2 siRNA

siRNA against the *EZH2* gene (Thermo Fisher Scientific, assay ID s4916; 5′‐GCUGACCAUUGGGACAGUATT‐3′) and negative control siRNA (Thermo Fisher Scientific, assay ID AM4611) were transfected into SBC3 with moderately high EZH2 expression and SBC5 cells with highest EZH2 expression, using Lipofectamine™ RNAiMAX Transfection Reagent (Thermo Fisher Scientific) based on the manufacturer's protocol.

### 2.6 Comprehensive miRNA expression analysis

We used Affymetrix GeneChip™ miRNA 4.0 Array (Affymetrix, Thermo Fisher Scientific, Santa Clara, CA, USA) to compare miRNA expression between SCLC cells transfected with *EZH2* siRNA‐transfected SCLC cells and those transfected with negative control siRNA (commissioned analysis by Filgen, Nagoya, Japan). The analysis was performed using SBC3 and SBC5 cell lines. GeneChips were scanned using the GeneChip Scanner 3000 7G (Affymetrix, Thermo Fisher Scientific) according to the Affymetrix GeneChip Command Console AGCC 4.0 User Manual. The data were analyzed with Microarray Data Analysis Tool Ver3.2 (Filgen). Changes in miRNA expression levels were calculated from the ratio of signal intensities of individual miRNAs in *EZH2* siRNA‐transfected cells to those in cells transfected with negative control siRNA. A change of more than two‐fold or less than 0.5‐fold in the expression levels was defined as significant. Among miRNAs whose expressions showed substantial changes in SBC3 and SBC5 cells similarly, we extracted a Homo sapiens‐derived miRNA that has been concerned with EZH2.

### 2.7 qPCR for miRNA

Following the manufacturer's protocol, cDNA was synthesized from total RNA isolated from cell lines using the TaqMan MicroRNA Reverse Transcription Kit (Thermo Fisher Scientific). Real‐time PCR with cDNAs was used by the TaqMan Universal PCR Master Mix (Thermo Fisher Scientific). U6 small nuclear RNA was employed as an internal control in quantitative PCR analysis. The experiments were conducted in triplicate. Each expression value was calculated with the ΔΔC_T_ method.

### 2.8 Target miRNA overexpression

To overexpress the target miRNA, 5 pmol of mirVana™ miRNA mimics for target miRNA (Thermo Fisher Scientific) were transfected using Lipofectamine RNAiMAX Transfection Reagent (Thermo Fisher Scientific). As a negative control, mirVana™ miRNA Mimic Negative Control #1 (Thermo Fisher Scientific, Cat #4464058) was used.

### 2.9 Western blot analysis

Cells plated into six‐well dishes were incubated in 2 mL of RPMI‐1640 medium for 48–72 h and harvested with CelLytic M Cell Lysis Reagent (Sigma‐Aldrich). The extracted proteins were heated at 95°C for 5 min and electrophoresed in 10% Mini‐PROTEAN® TGX™ Precast Gels (Bio‐Rad Laboratories Inc., Hercules, CA, USA). Proteins were then transferred onto polyvinylidene fluoride membranes (Millipore, Billerica, MA, USA) using iBlot (Invitrogen, Carlsbad, CA, USA). The membranes were immersed in TBS (pH 7.6) containing 5% skim milk and 0.1% Tween‐20 to block nonspecific binding and incubated with primary antibodies at 25°C for 1 h and then anti‐mouse or anti‐rabbit immunoglobulin G conjugate (Promega, Madison, WI, USA) (Table S1). The antibodies were diluted with Immuno‐Enhancer Reagent A (Wako, Osaka, Japan). The incubated membranes were washed and incubated with an ImmunoStar LD (Wako) for 1 min. Specific signals were scanned and analyzed using C‐DiGit Blot Scanner and Image Studio™ Software (LI‐COR Biosciences, Lincoln, NB, USA). GAPDH was employed as an internal control. Densitometry was carried out using ImageJ (National Institutes of Health, Bethesda, MD, USA). Each experiment was conducted in triplicate.

### 2.10 Cell proliferation assay

On the basis of the manufacturer's instructions, the cell proliferation assay was conducted with a CCK‐8 reagent (DOJINDO, Kumamoto, Japan). SBC3 and SBC5 cells were seeded into 96‐well dishes (6000 and 4000 cells per well, respectively) in RPMI‐1640 containing 10% FBS. Following this, 5 pmol of mirVana™ miRNA mimics for target miRNA (Thermo Fisher Scientific) or mirVana™ miRNA Mimic Negative Control #1 (Thermo Fisher Scientific, Cat #4464058) as a negative control was transfected using Lipofectamine RNAiMAX Transfection Reagent (Thermo Fisher Scientific). CCK‐8 reagent (10 μL) was added to each well, and cells were incubated at 37°C for 2.5 h. Absorbance at 450/620 nm was measured at 24, 48, 72, and 96 h. All assays were performed in triplicates.

### 2.11 Luciferase assay

Luciferase assay identified the *girdin* gene as a target of miR‐4448. mirVana™ miRNA mimic for miR‐4448 (Thermo Fisher Scientific, Cat #4464066; 5′‐ AGGAGUGACCAAAAGACAAGAGUGCGAGCCUUCUAUUAUGCCCAGACAGGGCCACCAGAGGGCUCCUUGGUCUAGGGGUAAUGCCA −3′) was co‐transfected with either the customized pEZX‐MT06 vector containing 3362 bp of the 3′UTR of the *girdin* gene (Cat No. HmiT059880‐MT06; Genecopoeia, Rockville, MD, USA) or pEZX‐MT06 (Cat No. CmiT000001‐MT06; Genecopoeia, Rockville, MD, USA) used as an empty vector into SBC5 cells following manufacturer's protocol. As a negative control, mirVana™ miRNA Mimic Negative Control #1 (Thermo Fisher Scientific, Cat #4464058) was employed. The light intensity of each cell was measured using a luminometer. The analysis was performed in triplicates.

### 2.12 Statistical analysis

Statistical analyses were performed with SPSS Statistics version 25 (IBM, Chicago, IL, USA). Students' *t*‐test or chi‐square test was employed to evaluate the correlations between EZH2 protein expression and each clinicopathological characteristic. Expression data for mRNA and miRNA using qPCR were log2 transformed. Differences in the expression levels in qPCR and Western blot analysis were assessed using a median test. All values are represented as mean ± SD; a *p* value <0.05 indicates statistical significance.

## 3 RESULTS

### 3.1 High EZH2 expression induces EMT in SCLC

We used immunohistochemical analysis to quantify the protein expression levels of EZH2 and E‐cadherin in 34 tumor tissues of patients with SCLC (Table 1). Compared to specimens from 34 stage‐matched patients with AD and 34 with SQ, the IRS of EZH2 was significantly higher in those from 34 SCLC tissues (*p* < 0.001). In contrast, the IRS for E‐cadherin was lower (*p* < 0.001). There was a correlation between high EZH2 and low E‐cadherin expressions, indicating EMT induction in SCLC.

**TABLE 1:** EZH2 and E‐cadherin protein expressions in lung cancer by immunohistochemical analysis.

		Histology			SCLC vs. AD	SCLC vs. SQ
		SCLC	AD	SQ	*p* Value	
Staining intensity grade (0–3), mean ± SD	EZH2	2.66 ± 0.61	1.41 ± 0.99	1.41 ± 0.86	<0.001	<0.001
	E‐cadherin	1.91 ± 1.03	2.76 ± 0.55	2.50 ± 0.79	<0.001	<0.001
Proportion of expressing cells (0–4), mean ± SD	EZH2	3.53 ± 0.83	2.29 ± 1.31	2.18 ± 1.22	<0.001	<0.001
	E‐cadherin	2.62 ± 1.46	3.88 ± 0.41	3.64 ± 0.81	<0.001	<0.001
Immunoreactive score (0–12), mean ± SD	EZH2	9.45 ± 3.28	4.45 ± 4.14	3.82 ± 3.33	<0.001	<0.001
	E‐cadherin	6.32 ± 4.50	10.91 ± 2.61	9.56 ± 3.45	<0.001	<0.001

Abbreviations: AD, adenocarcinoma; EZH2, enhancer of zest homolog 2; SCLC, small cell lung cancer; SD, standard deviation; SQ, squamous cell carcinoma.

**FIGURE 1:**
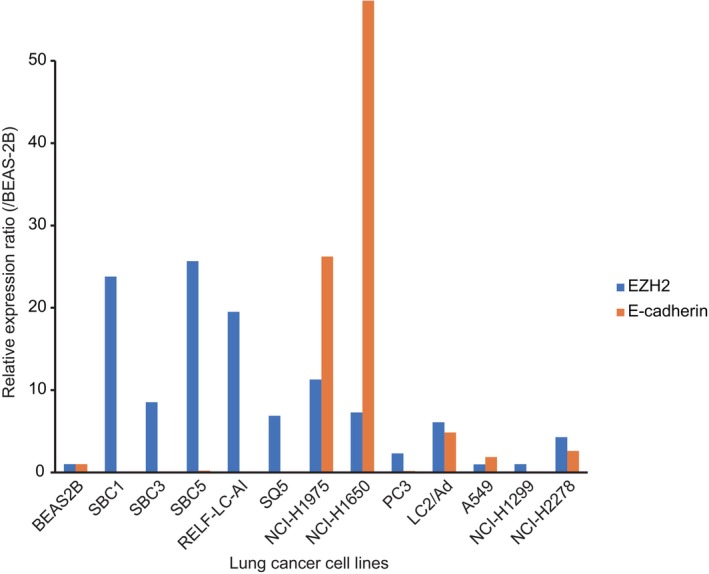
Relative expression levels of EZH2 and E‐cadherin in lung cancer cell lines compared to BEAS‐2B cell lines as a reference using qPCR analysis. Small cell lung cancer cell lines, SBC1, SBC3, and SBC5 cells, showed high *EZH2* and reciprocally low *E‐cadherin* expressions.

Subsequently, qPCR was performed to evaluate whether SCLC cell lines had high *EZH2* and low *E‐cadherin* expressions at the transcriptional level (Figure 1). Compared with BEAS‐2B cells, SBC1, SBC3, and SBC5 cells showed more than 8‐fold higher *EZH2* mRNA expression and less than 0.2‐fold lower *E‐cadherin* mRNA expression. Relative to the three SCLC cell lines, *EZH2* mRNA expression levels decreased in most NSCLC cells other than RERF‐LC‐AI and NCI‐H1975 cells, whereas those of *E‐cadherin* mRNA increased in most NSCLC cells other than RERF‐LC‐AI, Sq‐5 PC3, and NCI‐H1299 cells.

**FIGURE 2:**
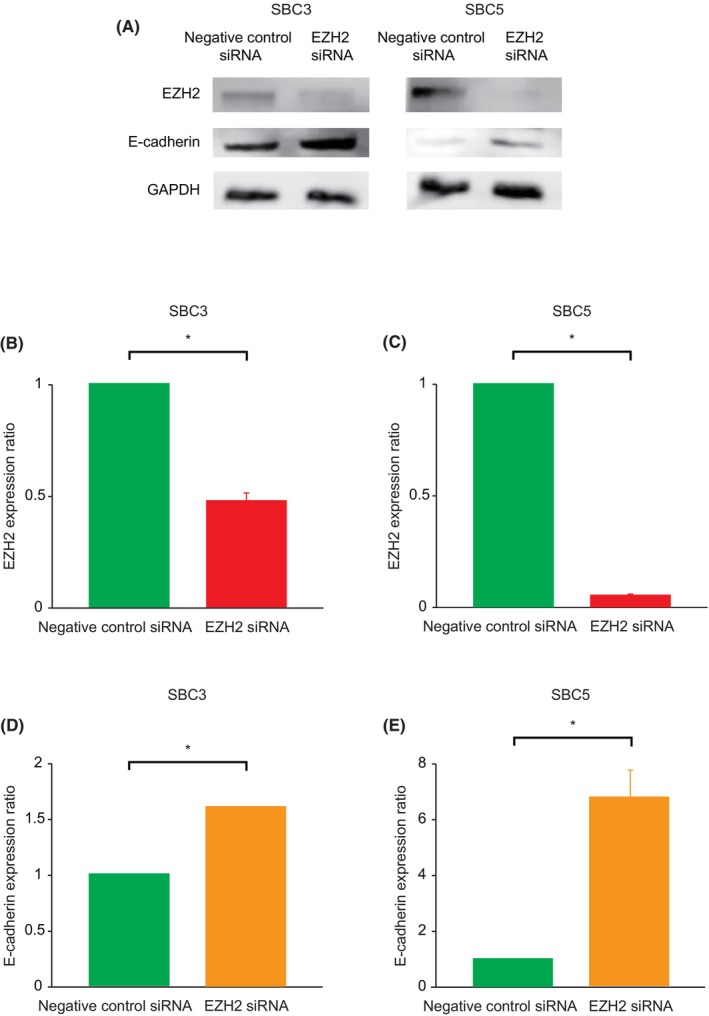
Protein expressions of EZH2 and E‐cadherin in small cell lung cancer cell lines by Western blot analysis. In SBC3 and SBC5 cells transfected with *EZH2* siRNA compared to those transfected with negative control siRNA, EZH2 expressions decreased (A, B, and C), whereas E‐cadherin expressions increased (A, D, and E). The uncropped blots are shown in Tables S1 and S2. *p < 0.05.

To confirm that EZH2 induced EMT, we knocked down *EZH2* mRNA using siRNA in SBC3 and SBC5 cell lines (Figure 2). Using Western blot analysis, each cell line with *EZH2* siRNA showed significantly reduced EZH2 protein expression and enhanced E‐cadherin protein expression (*p* < 0.05). These results suggested that EZH2 directly induced EMT, a characteristic of SCLC aggressiveness.

### 3.2 miR‐4448 suppresses EMT and inhibits SCLC cell proliferation

**FIGURE 3:**
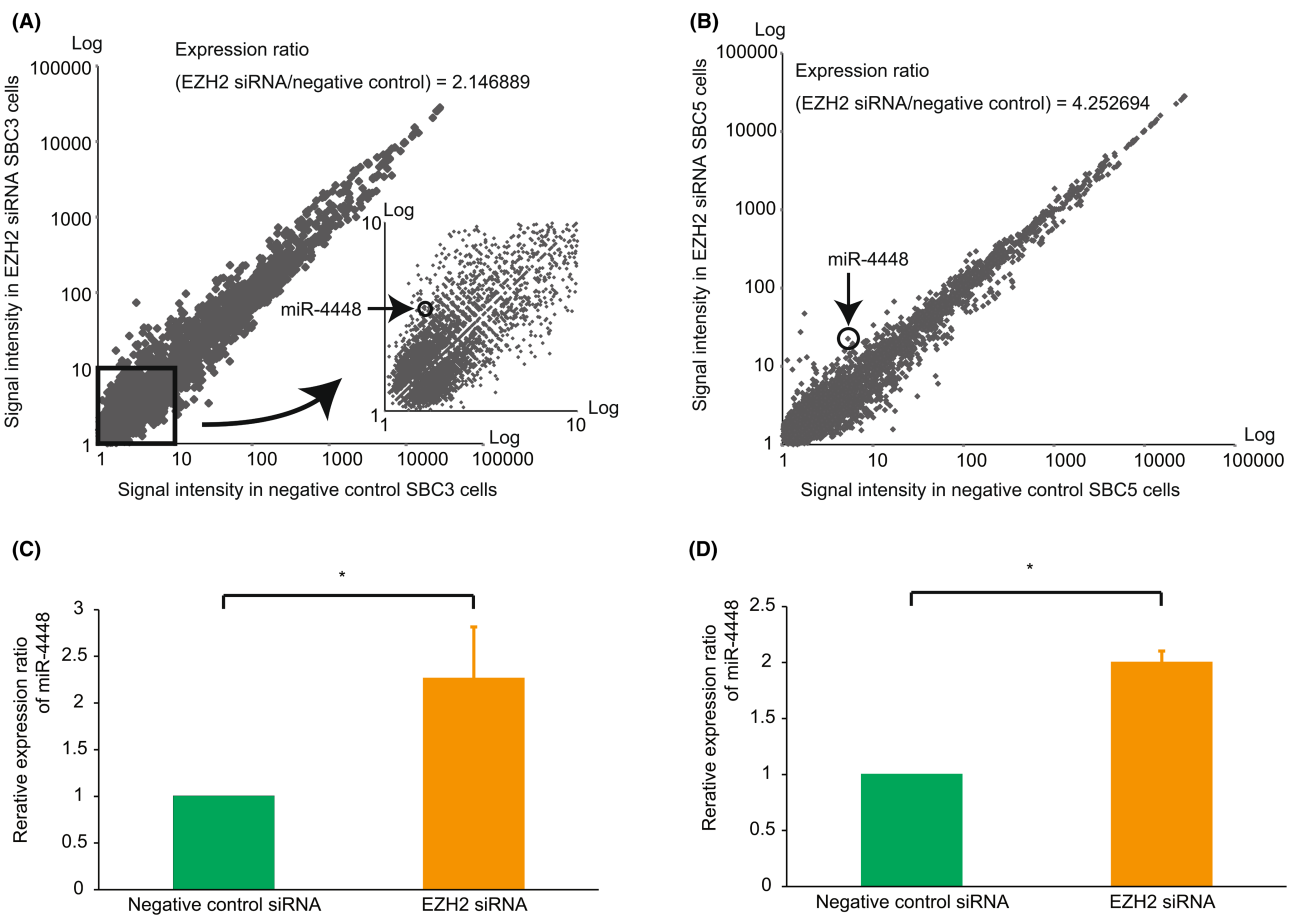
Comprehensive miRNA expression array and qPCR analysis for miR‐4448 in small cell lung cancer cell lines. Comprehensive miRNA expression array analysis using Affymetrix GeneChip™ miRNA 4.0 Array demonstrated that miR‐4448 expressions significantly increased in EZH2 siRNA‐transfected SBC3 (A) and SBC5 cells (B), compared to those transfected with negative‐control siRNAs. Data analysis was performed using Microarray Data Analysis Tool Ver3.2 (Filgen). qPCR analysis confirmed the array data (C and D). *p < 0.01.

Comprehensive miRNA expression analysis compared the miRNA expression profiles of *EZH2*‐knockdown SCLC cells with those of negative control siRNA‐transfected cells (Figure 3A,B). The miRNA array data in the present study were submitted to the GEO repository (accession no. GSE241822). Of the miRNAs whose expression levels showed significant differences between *EZH2*‐knockdown cells and negative control siRNA‐transfected cells (Table 2), we focused on miR‐4448, whose expression has been directly suppressed by EZH2 through promoter binding.^26^ qPCR confirmed the increased miR‐4448 expression in *EZH2*‐knockdown SBC3 and SBC5 cells (Figure 3C,D).

**TABLE 2:** miRNAs with >2‐fold or <0.5‐fold gene expression change by EZH2 siRNA in small cell lung cancer cells.

Accession ID	Species	miRNA	Sequence	Sequence Length	Fold change in SBC3	Fold change in SBC5
Increased expression						
MIMAT0025752	Gallus gallus	*miR‐6652‐5p*	UGGGUGUAUGUGGGACAGCUC	21	3.564	26.924
MIMAT0030000	Medicago truncatula	*miR‐7700‐5p*	GUGGAGUGUGGGACAGCUUGC	21	2.018	17.298
MIMAT0011304	Danio rerio	*miR‐2196*	CCUCUCUGUGCUGCCAUUUGGGAC	24	3.228	7.718
MIMAT0003276	Homo sapiens	*miR‐608*	AGGGGUGGUGUUGGGACAGCUCCGU	25	2.707	4.814
MIMAT0018967	Homo sapiens	*miR‐4448*	GGCUCCUUGGUCUAGGGGUA	20	2.147	4.253
MIMAT0016868	Homo sapiens	*miR‐4314*	CUCUGGGAAAUGGGACAG	18	3.523	3.469
MIMAT0024385	Pongo pygmaeus	*miR‐1273e*	GAGGCAGGAGAAUCGCUUG	19	2.994	2.955
MIMAT0015934	Pongo pygmaeus	*miR‐509‐3p*	UGAUUGGUACGUCUGCAGGUAG	22	5.028	2.671
MIMAT0007396	Glycine max	*miR‐1533*	AUAAUAAAAAUAAUAAUGA	19	2.801	2.536
MIMAT0000772	Homo sapiens	*miR‐345‐5p*	GCUGACUCCUAGUCCAGGGCUC	22	2.518	2.491
MIMAT0008111	Pan troglodytes	*miR‐345*	GCUGACUCCUAGUCCAGGGCUC	22	2.518	2.491
MIMAT0015846	Pongo pygmaeus	*miR‐345*	GCUGACUCCUAGUCCAGGGCUC	22	2.518	2.491
MIMAT0000402	Drosophila melanogaster	*miR‐310‐3p*	UAUUGCACACUUCCCGGCCUUU	22	2.010	2.370
MIMAT0008779	Drosophila sechellia	*miR‐310*	UAUUGCACACUUCCCGGCCUUU	22	2.010	2.370
MIMAT0008841	Drosophila simulans	*miR‐310*	UAUUGCACACUUCCCGGCCUUU	22	2.010	2.370
MIMAT0025684	Gallus gallus	*miR‐6591‐3p*	GCAGCCCGACGGACCGGCU	19	2.325	2.220
MIMAT0011710	Pristionchus pacificus	*miR‐2238d*	AAUGACAAGUGCAGAUGGCGAG	22	2.254	2.084
MIMAT0011631	Caenorhabditis remanei	*miR‐2231‐5p*	AACGGCAAAAACUACAGGUAGC	22	5.013	2.053
MIMAT0015041	Homo sapiens	*miR‐1260*	AUCCCACCACUGCCACCAU	19	2.246	2.050
MIMAT0024066	Pan troglodytes	*miR‐1260b*	AUCCCACCACUGCCACCAU	19	2.246	2.050
MIMAT0014054	Oryza sativa	*miR‐2923*	AGACAAAAAUAUAAAUAACAAA	22	2.838	2.043
MIMAT0009394	Mus musculus	*miR‐1931*	AUGCAAGGGCUGGUGCGAUGGC	22	2.878	2.042
Decreased expression						
MIMAT0019641	Petromyzon marinus	*miR‐4607*	GGGCCAUGGGGAAGAGGCCCGGCC	24	0.238	0.482
MIMAT0016179	Pongo pygmaeus	*miR‐1254*	AGUCUGGAAGCUGGAGCCUGCAGU	24	0.444	0.480
MIMAT0019936	Homo sapiens	*miR‐4778‐5p*	AAUUCUGUAAAGGAAGAAGAGG	22	0.474	0.388
MIMAT0027970	Mus musculus	*miR‐7033‐5p*	UCUCCAGGAGUCUGAGGGGCAGG	23	0.492	0.371
MIMAT0016313	Schistosoma japonicum	*miR‐3504*	GUGUGGUUGUCAGAAGGGGC	20	0.474	0.358
MIMAT0027928	Mus musculus	*miR‐7012‐5p*	AAGGAGAGGAGUUGGCAGGGACU	23	0.440	0.297

**FIGURE 4:**
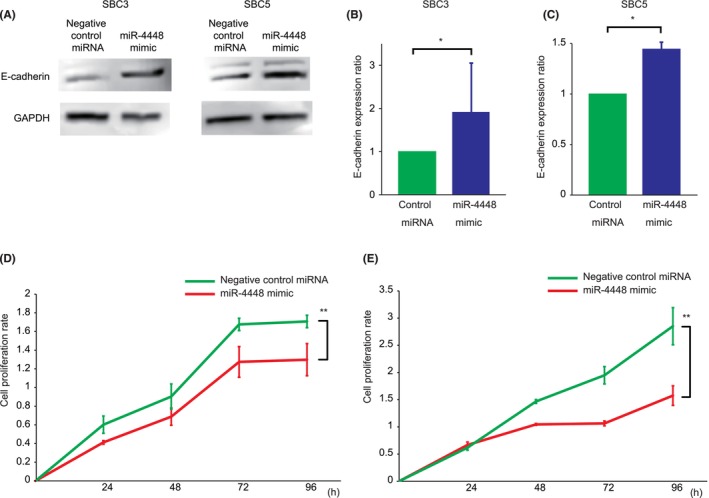
Protein expressions of E‐cadherin in small cell lung cancer cell lines transfected with miR‐4448 mimic by Western blot analysis and in vitro cell proliferation assay. E‐cadherin expressions increased in miR‐4448‐transfected SBC3 (A and B) and SBC5 cells (A and C), compared to those transfected with negative control miRNA. The uncropped blots are shown in Tables S1 and S2. miR‐4448‐transfected SBC3 (D) and SBC5 cells (E) reduced cell growth, compared to those transfected with negative control miRNA. *p < 0.05; *p < 0.01.

Next, miR‐4448 mimic (mirVana™ miRNA Mimic, Cat #4464066) or negative control miRNA mimic (mirVana™ miRNA Mimic Negative Control #1, Cat #4464058) was introduced into SBC3 and SBC5 cells. E‐cadherin protein expression was higher in SBC3 and SBC5 cells transfected with miR‐4448 mimic compared to those transfected with negative control miRNA (*p* < 0.05) (Figure 4A,B). These findings suggested that EZH2 suppressed miR‐4448 expression and induced EMT.

In cell proliferation assays, the proliferation rates of SBC3 and SBC5 cells transfected with miR‐4448 mimic were consistently lower than those of cells transfected with negative control miRNA (Figure 4C,D). Cell growth was significantly reduced after 96 h of transfection with miR‐4448 mimic. Further, induced miR‐4448 exhibited growth‐inhibiting activity in SCLC cells.

### 3.3 Girdin as a target of miR‐4448

**FIGURE 5:**
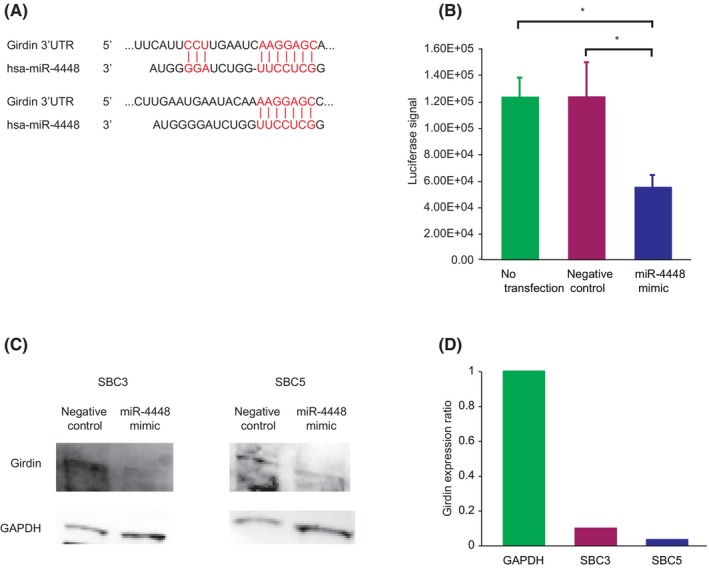
Girdin as a target of miR‐4448. (A) The putative binding site of miR‐4448 in the 3′UTR of the *girdin* gene. (B) Luciferase assay confirmed the 3′UTR of the *girdin* gene as a direct target of miR‐4448. (C) Representative image of Western blot analysis for Girdin expressions in small cell lung cancer cell lines transfected with miR‐4448 mimic. The uncropped blots are shown in Tables S1 and S2. (D) Girdin expressions in miR‐4448‐transfected SBC3 and SBC5 cells were attenuated, compared to those of GAPDH used as an internal control. *p < 0.05.

A previous study showed that EZH2 inhibition upregulated miR‐4448, leading to the repression of the *girdin* gene by binding to the 3′UTR (Figure 5A).^26^ Luciferase assay and Western blot analysis identified an association between miR‐4448 and Girdin. In the luciferase assay, miR‐4448 mimic‐transfected SBC3 cells reduced luciferase activity through the 3′UTR of the *girdin* gene compared to negative control miRNA‐transfected or non‐transfected cells (Figure 5B). Western blot analysis demonstrated that Girdin expression decreased in SBC3 and SBC5 cells transfected with miR‐4448 mimic compared to those transfected with negative control miRNA (Figure 5C,D). These results suggested that miR‐4448 negatively regulated Girdin as a target in SCLC.

### 3.4 Girdin suppression induces pAMPK via decreased pAkt, leading to pEZH2

**FIGURE 6:**
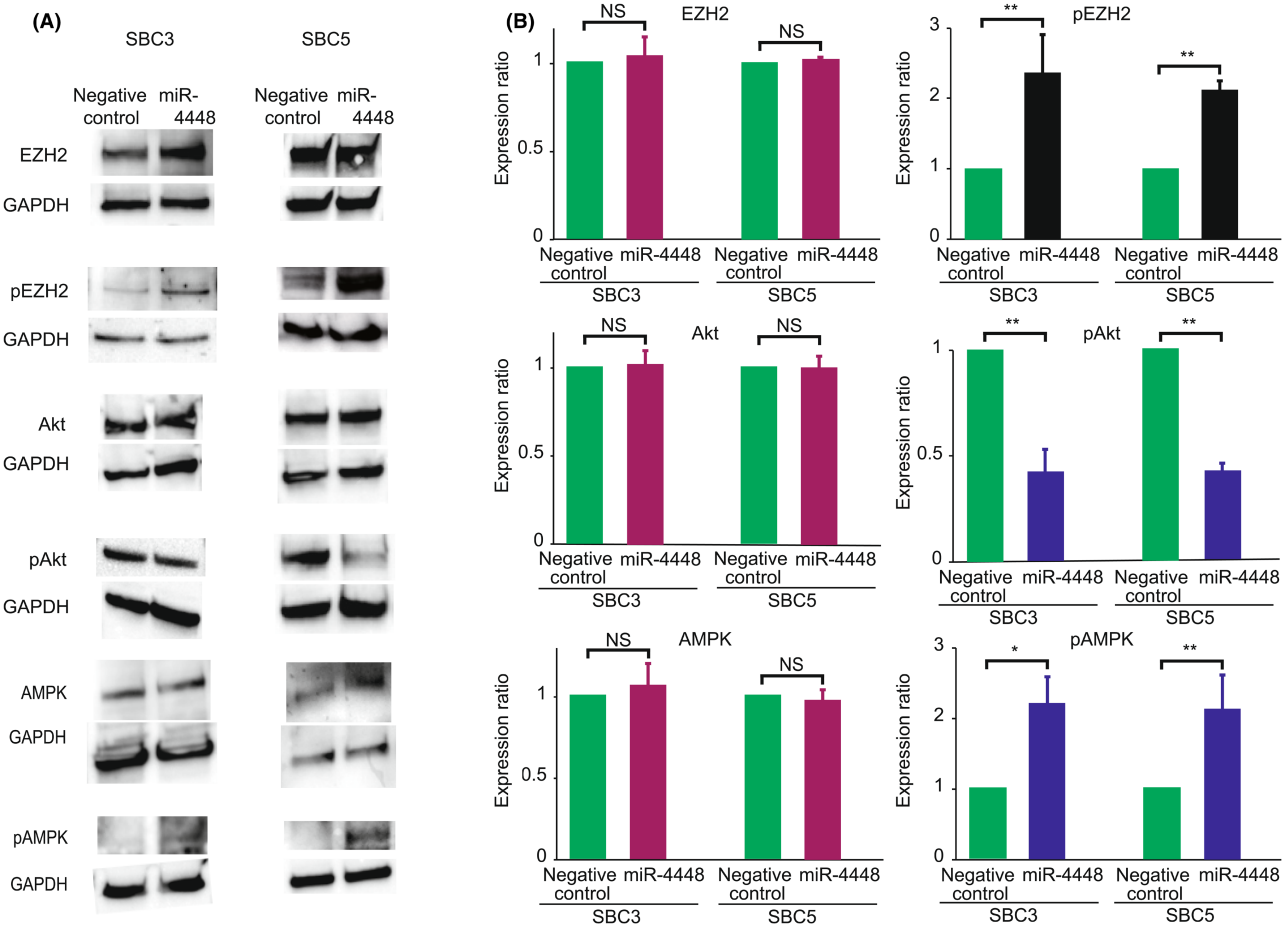
Western blot analysis of the Akt/AMPK pathway and EZH2. In SBC3 and SBC5 cells transfected with miR‐4448 mimic, pAkt expression was reduced, whereas pAMPK and pEZH2 expressions were induced, compared to those transfected with negative control miRNA. (A) Representative images of Western blot. The uncropped blots are shown in Tables S1 and S2. (B) Quantified data of protein expressions. *p < 0.05; **p < 0.01; NS, nonsignificant.

Girdin, a substrate of Akt, is considered to activate the PI3‐Akt pathway.^28^ Previous studies have shown that phosphorylated Akt (pAkt) at Ser473 and phosphorylated AMPK (pAMPK) at Thr172 and 183 constituted a negative feedback loop.^29^, ^30^, ^31^ Wan et al. reported that AMPK activation induced phosphorylated EZH2 (pEZH2) at Thr311, suppressing tumorigenesis.^32^ We investigated whether miR‐4448‐mediated *girdin* gene suppression modulated the expressions of pAkt at Ser473, pAMPK at Thr172 and 183, and pEZH2 at Thr311. Compared to negative control‐transfected and non‐transfected cells, pAkt at Ser473 reduced in miR‐4448‐transfected cells, where Girdin expression was suppressed. In contrast, pAMPK at Thr172 and 183 and pEZH2 at Thr311 were induced in miR‐4448‐transfected cells (Figure 6). These results are consistent with previous findings.

## 4 DISCUSSION

The present study revealed that miR‐4448 prevented EZH2‐mediated EMT and tumorigenesis through Girdin suppression and modification of the Akt/AMPK pathway in SCLC. Roche et al. have reviewed EMT induction through EZH2 in NSCLC.^7^ In previous studies, miRNAs have impacted EZH2 function in NSCLC.^33^, ^34^ Other reports have shown miR‐4448 as an EZH2 regulator and the miR‐200 family and miR‐205 as EMT modulators in NSCLC.^26^, ^35^, ^36^ In addition to the importance of miRNA‐mediated EMT in NSCLC, high EZH2 expression has been reported in cell lines and clinical specimens of SCLC.^24^, ^37^, ^38^ In the current study, SCLC was characterized by high EZH2 expressions and reciprocally low E‐cadherin expressions, indicating that EZH2 contributed to EMT in SCLC.

Furthermore, Uddin et al. reported that multiple miRNAs were involved in SCLC pathogenesis.^39^ Such evidence raised the hypothesis that miRNAs may determine EZH2‐mediated EMT modulation in SCLC. However, to our knowledge, no reports have shown direct correlations between miRNAs and EZH2 in SCLC. Our findings suggested that miR‐4448 suppressed EZH2‐induced EMT in SCLC.

**FIGURE 7:**
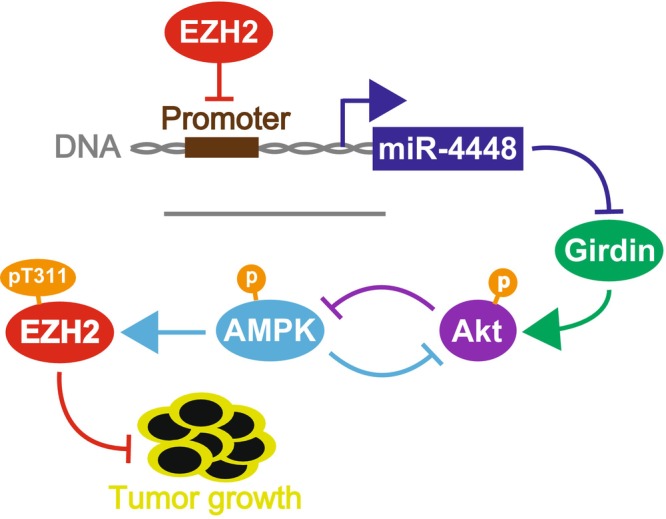
The scheme of the potential mechanism underlying tumor growth inhibition through the miR‐4448/Girdin/Akt/AMPK/EZH2 axis. EZH2 targets the promoter of miR‐4448, which binds to the 3′UTR of the girdin gene and disrupts its expression. Girdin suppression reduces pAkt at Ser473, inducing pAMPK at Thr172 and 183 and pEZH2 at Thr311. Consequently, miR‐4448 prevents tumor growth through pEZH2 at Thr311.

Previously reported studies have shown that miR‐4448 is related to a response to radiation exposure, chemoresistance to breast cancer, and self‐renewal capability.^40^, ^41^ Hibino et al. have demonstrated that EZH2, bound to the miR‐4448 promoter region, suppresses its expression.^26^ Additionally, the report showed that EZH2 inhibition enhanced miR‐4448 expression and attenuated Girdin expression, suggesting that miR‐4448 suppresses the *girdin* gene by binding to its promoter region. We confirmed that miR‐4448 repressed Girdin expression in SCLC cells, and luciferase assay revealed that miR‐4448 targeted the *girdin* gene. Therefore, miR‐4448‐mediated Girdin knockdown was also deemed to underlie miR‐4448‐induced E‐cadherin upregulation in Figure 4. Girdin, as a substrate of Akt, promotes cell migration, tumor growth, and metastasis through the PI3K/Akt signaling pathway.^42^, ^43^ pAkt is known to regulate pAMPK negatively.^29^, ^30^, ^31^ Wan et al. showed that AMPK activation induces pEZH2 at Thr311, preventing PRC2‐mediated oncogenic function.^32^ Given such evidence, the current study demonstrated that miR‐4448 reduced pAkt expression by targeting Girdin, leading to induced pAMPK and pEZH2 at Thr311. Previous studies suggested Girdin as a potential target of miR‐4448, Akt‐mediated AMPK modulation, and AMPK‐induced pEZH2 at Thr311 independently.^28^, ^32^, ^36^, ^43^ However, no studies have reported that miR‐4448 targeted Girdin and inhibited its expression directly. Furthermore, the miR‐4448/Girdin/Akt/AMPK/EZH2 axis has yet to be advocated. Together with our findings and previous reports, miR‐4448 inhibited Girdin, attenuating phosphorylation of Akt, a negative regulator of AMPK, resulting in enhanced pAMPK.^26^, ^29^, ^30^, ^31^, ^42^, ^43^ Consequently, activated pAMPK induced pEZH2 at Thr311, suppressing SCLC tumorigenesis and EMT.^32^ miR‐4448 The novel miR‐4448/Girdin/Akt/AMPK/EZH2 axis might be a potential pathway for EZH2‐mediated EMT and tumorigenesis (Figure 7).

However, the present study has potential limitations. First, human tissue samples were used to analyze EZH2 and E‐cadherin expressions. In contrast, in vitro studies elucidated the tumorigenic mechanism of the miR‐4448/Girdin/Akt/AMPK/EZH2 axis. In vivo experimental studies using mice SCLC models transfected with miR‐4448 may ensure the molecular dynamics intravitally. Second, we employed SBC3 and SBC5 as SCLC cells widely used for in vitro studies. Recently, SCLC was classified into four subtypes based on expressional patterns of transcription regulators.^44^ According to this classification, SBC3 and SBC5 belong to the non‐neuroendocrine type. However, these cells are representative SCLC cell lines and have commonly been used for SCLC research in vitro models. Accumulating pathological evidence based on the molecular subtype may provide an adequate answer to an appropriate SCLC model. Third, the present study focused on miR‐4448 and EZH2, particularly miR‐4448‐mediated tumorigenesis, consequently identifying the miR‐4448/Girdin/Akt/AMPK/EZH2 axis. Thus, future studies will be warranted to elucidate further functions of individual molecules in the axis: Girdin, Akt, and AMPK.

In summary, EZH2 suppressed the expression of miR‐4448, which directly inhibited the *girdin* gene, thereby preventing EZH2‐induced EMT and tumorigenesis through the Akt/AMPK pathway in SCLC. Previous reports have demonstrated the associations between Girdin and Akt, Akt and AMPK, and AMPK and EZH2.^29^, ^30^, ^31^, ^32^, ^42^, ^43^ However, the miR‐4448/Girdin/Akt/AMPK/EZH2 axis through miRNA and its tumorigenic mechanism in SCLC has not been shown. Given the poor outcome of SCLC in clinical practice, the current study may provide a cue for SCLC disease control using miR‐4448‐mediated EZH2 suppression through the Girdin/Akt/AMPK pathway.

## AUTHOR CONTRIBUTIONS

**Nobuyuki Koyama:** Conceptualization (lead); data curation (lead); formal analysis (lead); funding acquisition (lead); investigation (lead); methodology (lead); project administration (lead); writing – original draft (lead). **Yuichi Ishikawa:** Methodology (equal); supervision (lead); validation (lead); writing – review and editing (lead). **Hiromitsu Ohta:** Data curation (equal); investigation (equal). **Takuya Aoki:** Validation (equal); writing – review and editing (equal). **Hiroyuki Kyoyama:** Data curation (equal); investigation (equal). **Kazutetsu Aoshiba:** Methodology (equal); supervision (equal); validation (equal); writing – review and editing (equal). **Kazutsugu Uematsu:** Methodology (equal); supervision (equal); validation (equal); writing – review and editing (equal).

## FUNDING INFORMATION

KAKENHI grants‐in‐aid for Scientific Research (C) from the Japan Society for the Promotion of Science (JSPS) (grant no. 26461195); Project Research 2 from the Smoking Research Foundation (grant no. 2019T0 express13).

## CONFLICT OF INTEREST STATEMENT

Yuchi Ishikawa is a consultant for Fujirebio Inc. Other authors have no conflict of interest.

## ETHICS STATEMENT

The institutional review board of Jichi Medical University Saitama Medical Center approved the immunohistochemical analysis using clinical samples (No. 14–13). The institutional review board of Jichi Medical University Saitama Medical Center waived the informed consent for the noninvasive retrospective study. All experiments were implemented following approved guidelines and regulations.

## Supporting information


Table S1.

Table S2.

## Data Availability

The datasets employed in the current study are available from the corresponding author upon reasonable request. The miRNA array data in the present study were submitted to the GEO repository (accession no. GSE241822).
