# Supplementary material for: miR‐4448/Girdin/Akt/AMPK axis inhibits EZH2‐mediated EMT and tumorigenesis in small‐cell lung cancer
Source: Cancer Med. 2024 Oct 14;13(19):e70093. doi: 10.1002/cam4.70093 (PMC11476246; doi:10.1002/cam4.70093)
Supplement: Supplementary file 1 — Table S1. [file CAM4-13-e70093-s002.docx]

| TABLE S1. Antibody information on western blot analysis | | |
| --- | --- | --- |
| Antibodies | Manufacture | Catalog number |
| Rabbit polyclonal Akt | Cell Signaling Technology (Danvers, MA, USA) | #9272 |
| Rabbit olyclonal phospho-Akt (Ser473) | Cell Signaling Technology | #9271 |
| Rabbit polyclonal AMPKα1 | GeneTex (Irvine, CA, USA) | GTX112999 |
| Rabbit polyclonal phospho-  AMPKα1 (Thr183) + AMPKα2 (Thr172) | GeneTex | GTX130429 |
| Rabbit monoclonal E-cadherin | Cell Signaling Technology | #3195 |
| Mouse monoclonal EZH2 | Cell Signaling Technology | #3147 |
| Rabbit polyclonal phospho-EZH2 (Thr311) | Cell Signaling Technology | #27888 |
| Rabbit monoclonal GAPDH | Cell Signaling Technology | #3683 |
| Mouse monoclonal Girdin | Santa Cruz Biotechnology (Dallas, TX, USA) | sc-393757 |
| Abbreviations: AMPK; AMP-activated protein kinase; EZH2; enhancer of zeste homolog 2 | | |
