# Supplementary material for: miR‐4448/Girdin/Akt/AMPK axis inhibits EZH2‐mediated EMT and tumorigenesis in small‐cell lung cancer
Source: Cancer Med. 2024 Oct 14;13(19):e70093. doi: 10.1002/cam4.70093 (PMC11476246; doi:10.1002/cam4.70093)
Supplement: Supplementary file 2 — Table S2. [file CAM4-13-e70093-s001.docx]

| TABLE S2. Primer sequences in quantitative real-time RT-PCR | | |
| --- | --- | --- |
| Gene | Forward primer | Reverse primer |
| E-cadherin | 5'-CGGAACCGCTTCCTTCATAG-3' | 5’-TGAGTGTCCCCCGGTATCTT-3’ |
| EZH2 | 5’-GTGACCCTGACCTCTGTCTTACTT-3’ | 5’-CCCTCTTCTGTCAGCTTCATCT-3’ |
| GAPDH | 5’-CCTCAACGACCACTTTGTCA-3’ | 5’-TTACTCCTTGGAGGCCATGT-3’ |
| Abbreviations: EZH2, enhancer of zeste homolog 2 | | |
